# Brain Tumor Segmentation Using Deep Learning on MRI Images

**DOI:** 10.3390/diagnostics13091562

**Published:** 2023-04-27

**Authors:** Almetwally M. Mostafa, Mohammed Zakariah, Eman Abdullah Aldakheel

**Affiliations:** 1 Department of Information Systems, College of Computer and Information Sciences, King Saud University, P.O. Box 51178, Riyadh 11543, Saudi Arabia; almetwaly@ksu.edu.sa; 2 Department of Computer Science, College of Computer and Information Science, King Saud University, P.O. Box 51178, Riyadh 11543, Saudi Arabia; mzakariah@ksu.edu.sa; 3 Department of Computer Sciences, College of Computer and Information Sciences, Princess Nourah bint Abdulrahman University, P.O. Box 84428, Riyadh 11671, Saudi Arabia

**Keywords:** brain tumor detection, MRI images, DL, CNN model, image classification

## Abstract

Brain tumor (BT) diagnosis is a lengthy process, and great skill and expertise are required from radiologists. As the number of patients has expanded, so has the amount of data to be processed, making previous techniques both costly and ineffective. Many academics have examined a range of reliable and quick techniques for identifying and categorizing BTs. Recently, deep learning (DL) methods have gained popularity for creating computer algorithms that can quickly and reliably diagnose or segment BTs. To identify BTs in medical images, DL permits a pre-trained convolutional neural network (CNN) model. The suggested magnetic resonance imaging (MRI) images of BTs are included in the BT segmentation dataset, which was created as a benchmark for developing and evaluating algorithms for BT segmentation and diagnosis. There are 335 annotated MRI images in the collection. For the purpose of developing and testing BT segmentation and diagnosis algorithms, the brain tumor segmentation (BraTS) dataset was produced. A deep CNN was also utilized in the model-building process for segmenting BTs using the BraTS dataset. To train the model, a categorical cross-entropy loss function and an optimizer, such as Adam, were employed. Finally, the model’s output successfully identified and segmented BTs in the dataset, attaining a validation accuracy of 98%.

## 1. Introduction

A tumor is created when aberrant cells divide out of control, generating a mass that might impair the tissue or organ’s ability to function normally [1,2]. The genesis, foundation, and cell types of tumors are distinct characteristics. The brain shows early stages of tumors mostly in the cerebrum region, although secondary tumors enter the brain from other parts of the body [2]. Cancerous tumors are classified as malignant (high-grade) or benign (low-grade), respectively. A malignant brain tumor (BT) develops faster than a benign BT and is more likely to infect surrounding tissues [3]. Consequently, a primary malignant BT has an unfavorable prognosis and significantly lowers cognitive function and quality of life [4]. Despite the fact that medical technology is fairly sophisticated nowadays, it remains an issue that some diseases are difficult for doctors to identify early. BT is diagnosed by doctors using brain tomography and magnetic resonance imaging (MRI) scans [5]. These images from several patients have been gathered. For the benefit of patients, a technique to detect the condition early is essential. Cancerous BTs are extremely hazardous and can be deadly [6]. Chemotherapy and radiation are two treatment options for the condition [7,8]. The most common form of treatment for this condition is surgery. The tumor’s pressure on the brain is the cause of this surgery.

BTs are tumors that originate in the skull; therefore, depending on how much pressure is added, they may present with a number of symptoms [9]. The most significant symptoms include severe headaches, vomiting, and nausea. Patients might suffer from circulatory system abnormalities and potentially be paralyzed as a result of the condition. Additionally, depending on the part of the brain that is injured, different symptoms might manifest. These include speech difficulties, hearing, numbness, and vision and gait disturbances, as well as weakness and numbness [10]. The precise cause of BTs is unknown. However, it is unquestionably true that BTs may be observed in people of various ages [11]. As the world’s population ages, more and more people will encounter this disease [12,13]. The condition is extremely deadly, meaning BT diagnostic research is a particularly active area of study [14]. The use of deep learning (DL) architecture, particularly convolutional neural networks (CNN), partially tackles this issue. A CNN model is a hybrid of a feature extractor and a classifier. Many people are currently interested in utilizing CNNs to create computer-aided design (CAD) systems. Applications for CAD that use CNNs have had great success and produced impressive outcomes. The classification of breast cancer as benign or malignant using CNN on histological images has recently been studied [15]. Additionally, multiclass issues are included in the paper [15]. As part of the diagnosis of lung diseases [13,16], this work formulates and explains tissue characterization. A wide range of difficulties is encountered while designing and putting automated CAD systems into use. The first factor affecting CNN’s feature extraction capability is the architecture of its layers. The second factor determining the generalization capacity of CNN on the data is the quantity of training data that is available. The precision with which CNN classifies test data is determined by the aforementioned elements. Therefore, many approaches to the categorization difficulties are taken into consideration.

Another approach to overcome the practical difficulties is to increase classification accuracy by fusing CNN features with tested classifiers. Several applications have successfully tested the ensemble configuration of CNN for feature extraction and a separate classifier. A support vector machine (SVM) employed the in-depth characteristics that were generated from hyperspectral images to categorize the data [17]. Performance was improved over using just the CNN model with the Softmax classifier when SVM and CNN were used in an ensemble [18]. In the area of bioinformatics, combining two machine learning (ML) methods has been effective [19]. Several researchers have studied the detection of BTs. In their research [20,21], they made use of the BraTS 2015 data collection. The approach they suggested, according to them, had encouraging outcomes. An automated approach that determines whether or not the brain contains a tumor from MRI images was proposed by Amin et al. The trials employed local datasets, and the best accuracy rate recorded was 98.2% [21]. A color-based segmentation to find BT was suggested by Wu et al. K-means clustering is the technique used in this strategy. To help pathologists pinpoint the precise size and location of the lesion, they asserted that the approach could correctly segment MRI-BT images [22]. For the purpose of detecting BTs, Irmak et al. [22] suggested a model. This model was built on a genetic algorithm. With the use of a genetic algorithm, tumor pixels on MRI images may be found [22].

**Figure 1:**
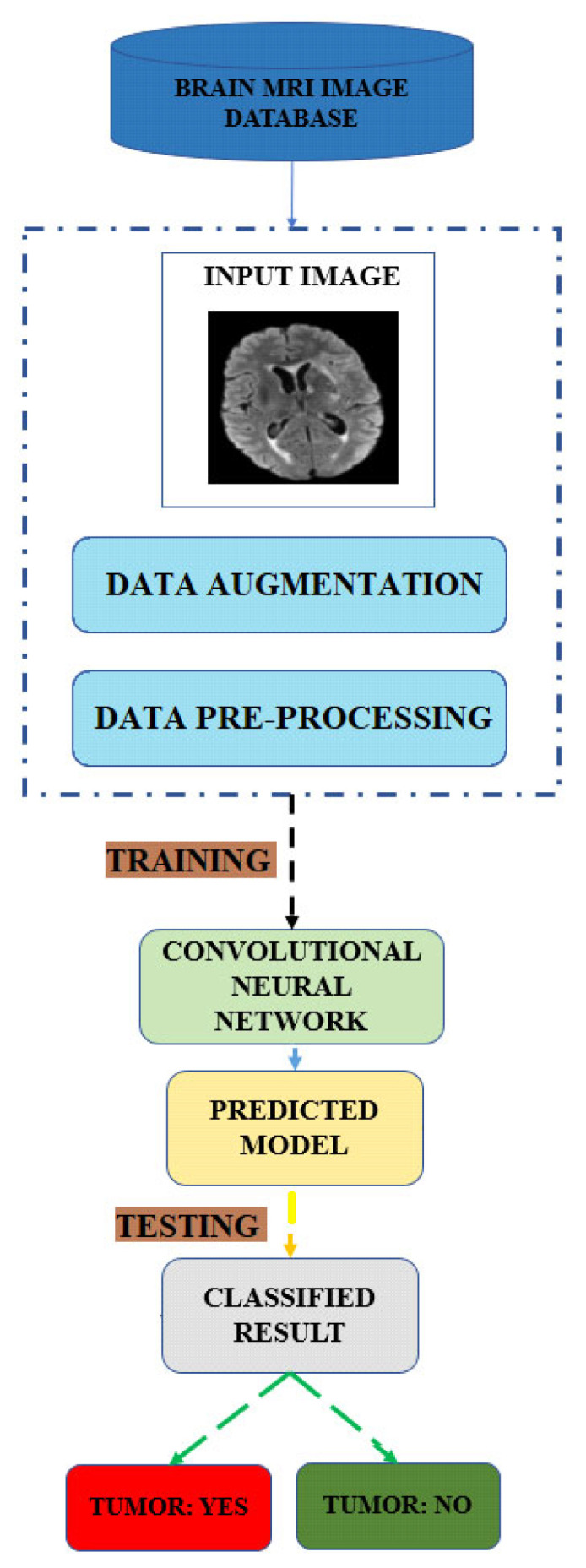
Proposed framework of deep learning method for BT detection.

Figure 1 shows the overall proposed framework for brain tumor identification using deep learning methods. The framework consists of several stages, starting with retrieving brain images from a database. After feature extraction, the data are augmented and preprocessed to improve the dataset. Data augmentation is a technique that generates new training data from existing data by adding variations to the existing images, such as flipping, rotating, or scaling. Preprocessing involves techniques such as normalization, denoising, and cropping. The training and testing stages constitute the main part of the proposed framework. DCNN is used in both stages. In the training phase, the feature extraction involves extracting relevant features from the brain images using U-Net sampling. The DCNN model is trained on a dataset to learn the patterns and features that are associated with brain tumors. The DCNN model forecasts the model parameters that minimize the prediction error. In the testing phase, the DCNN with the U-Net sampling model is used to classify the test data into tumor or non-tumor classes. Finally, the proposed framework predicts whether there is a brain tumor present or not based on the output of the proposed model.

In this study, we suggest an MRI image-based BT classification technique that is completely automated. The specified objective is to categorize three different forms of cancers in brain scans, including gliomas and pituitary tumors, using a three-class classification problem. These three BT types are the most prevalent [23]. A DCNN with a U-Net sampling model is used for classification, as well as to extract image features. The open dataset from Fig share [24,25] is used in the evaluation. Our work is motivated by the following factors: First, greater accuracy in the categorization problem involving meningiomas, gliomas, and endocrine tumors is expected. Medical professionals’ treatment regimens would benefit from a precise, computer-aided automatic classification approach for the three different types of tumors. Second, recent classification challenges that employed DL techniques and reliable classifiers were successful in producing highly accurate results. Medical imaging data are hard to attain, which is the third issue. To overcome this practical restriction, advanced design techniques are needed. The following are the contributions made by this work:

- To extract characteristics from brain MRI images containing tumors, a CNN model was created;
- On datasets containing medical images, it was discovered that the CNN layout produced better classification results;
- The feature maps are produced after numerous convolutional layers have extracted features from the input images;
- The BraTS dataset was used to create a model with 98% overall accuracy;
- Regarding computational complexity, a comparison between the proposed approach and a transfer learning-based strategy is given.

This article is organized as follows: Section 1 of the article contains the introduction. Section 2 contains a list of the pertinent work. The dataset is relevant to Section 3. The proposed technique is discussed in the following sections. Section 5 and Section 6 are connected to the paper’s results section and discussion section. The conclusion is described in Section 7.

## 2. Literature Review

Although CNN has been used extensively to solve a variety of issues in diverse fields [1,2,4,7,8], its performance is impressive when processing images for use in health care. Numerous researchers have suggested using CAD to diagnose illnesses. A neutrosophic CNN is being studied for BT detection [12,13,14]. In this hybrid approach, a CNN is utilized to extract features, whereas SVM and K-nearest neighbors (KNN) are applied for classification. Only 160 images, 60 of them negative and 60 of them good, were used for the system’s training and testing. The suggested method yields a 96.73% accuracy using fivefold cross-validation. Image enhancement technologies such as contrast augmentation and midrange stretching are used in the early phases of the process to increase the quality of MRI scans. After the tumor contour was produced using a semiautomatic method by [14], 71 features were built utilizing the intensity profile, the resulting co-occurrence matrix with updated values, and also the Gabor functions. Skull stripping is a frequent preprocessing step in traditional discriminative techniques [13,14] due to drawbacks such as parameter selection or the requirement for prior information on the images as well as lengthy computation durations. According to [16], a multigrade BT classification system was built using DL. This technique segments the tumor using the CNN model; however, the accuracy and sensitivity of the findings are constrained by the CNN model’s shortcomings [17,18,19]. Another study’s findings [20] indicate that both handmade and DL characteristics may be used to identify BTs.

This work [20] created five clinical multiclass datasets. To enhance BT classification performance using MRI images, they developed a transfer learning-based CNN. Six other ML classification methods, including decision tree (DT) [26], KNN [27], Naïve Bayes (NB) [28], linear discrimination (LD), and SVM [5], were evaluated with the indicated CNN model [29]. When considering the five different sorts of multiclass classification BT datasets, the suggested CNN-based (DL) model approach outperforms the six different kinds of ML model techniques. The CNN-based AlexNet model used three different cross-validation techniques, K2, K5, and K10, to attain mean accuracy rates for the five classes of 86.24, 94.63, 95.84, 97.54, and 100%, respectively. To discriminate among three different types of BT, including gliomas, meningiomas, and pituitary tumors, ref. [22] developed a CNN approach for a three-class categorization. To extract features from brain MRI data, they applied a pretrained GoogleNet. The retrieved characteristics are identified using proven-based classifiers. With an average classification accuracy of 98%, the proposed technique exceeds previous approaches. The performance measures used in the study include specificity, F-score, recall, and the area under curve (AUC). The research’s findings indicate that when there are not many medical images, transfer learning techniques are a highly significant strategy.

According to [24], CNN had an overall accuracy of 91.3% and a recall of 89%, 82%, and 99% in the diagnosis of meningioma, glioma, and pituitary tumors, respectively. To classify the various forms of BT from MRI image slices, DL architecture uses 2D convolutional neural networks. Data collecting, data preprocessing, pre-modeling, model optimization, and hyperparameter tweaking are some of the approaches used in this work. Additionally, the entire dataset was subjected to a 10-fold cross-validation to test the model’s generalizability [30]. The approach used in this study is based on Hough voting, a method that allows automated localization and segmentation of the relevant anatomies. Additionally, a segmentation approach based on learning techniques was employed, which is reliable, multiregional, adaptable, and suitable for usage with various modalities. To forecast the outcomes, several data dimensionalities (2D, 2.5D, and 3D) and training data volumes are used. To analyze the image, a CNN is coupled with voxel-wise and Hough voting classification, as well as effective patch-wise assessment [8].

The most current use of MRI in computer vision for information was described by [31]. The rapid identification and localization of cerebral tumors are made possible by MRI. With seven distinct, multiple groups of tumors and one showing a healthy cerebrum, we want to divide cerebrum scans into eight classes. It has been verified that the suggested classification strategy’s univariate method works. A novel architecture for BT classification was created using the RCNN (region-based convolutional neural network) method and evaluated two publicly available datasets from Kaggle and Fig share [32,33,34,35]. To speed up processing compared to a conventional RCNN structure, the authors presented a technique for BT detection. They employed a two-channel CNN, a low-complexity framework that increases accuracy by 98.21%, to classify between melanoma and healthy tumor MRI images. Then, a melanoma MRI dataset that had been classified from a previous phase was used to detect cancer locations using this architecture as a feature extractor in an RCNN. Finally, boxes encircled the tumor site. Pituitary and meningioma tumors have both been treated with this strategy. In comparison to cutting-edge methods, their methodology could reach a short execution time with an overall confidence level of 97.956%.

The segmentation of medical images is another subject that modern scientists study. The U-Net [36] was used in a publication [35] to demonstrate fully automatic segmentation of brain tumors from MR images. The quicker RCNN network was used to categorize and segment tumors by [37], who also studied the processing of MRI images. The localization of multiple sclerosis lesions in MRI images was also accomplished by [38] using a customized CNN network.

Table 1 presents a comprehensive overview of the reference names, datasets, techniques utilized, and the corresponding results of the brain tumor identification studies.

## 3. Dataset Collection

### 3.1. BraTS Dataset

The MRI scans of BTs are part of the BraTS collection, which was assembled from data from several institutes. Hospitals, colleges, and research organizations are some of the institutions that contributed to the dataset. The patients whose MRI scans were used to create the dataset gave their informed agreement to have their medical information used for research. The BraTS dataset was developed as a standard for designing and assessing BT segmentation and diagnostic algorithms. Annotated MRI images from 335 different patients were included in the most recent release [40] of the dataset, which is updated yearly. The data were preprocessed to standardize the size, intensity values, and other imaging characteristics. The MRI scans were taken utilizing various MRI equipment and procedures.

### 3.2. Data Description

A collection of BT MRI images makes up the BraTS dataset. In the realm of medical image analysis, it is used to create and test methods for BT segmentation and diagnosis. The BraTS dataset is a benchmark dataset for BT detection algorithms assessment and is frequently utilized in the research community. The collection contains MRI images using several modalities, including fluid-attenuated inversion recovery (FLAIR), T2-weighted, and T1-weighted with contrast. Additionally, it has annotations for the various tumor areas (peritumoral edema, enhancing tumor, and necrosis). Each voxel in the annotations is identified as either belonging to a tumor area or as being the backdrop using binary masks. Many algorithms for BT segmentation and diagnosis are developed and tested using the BraTS dataset. With the BraTS dataset, there are five different MRI image data formats available.

**Figure 2:**
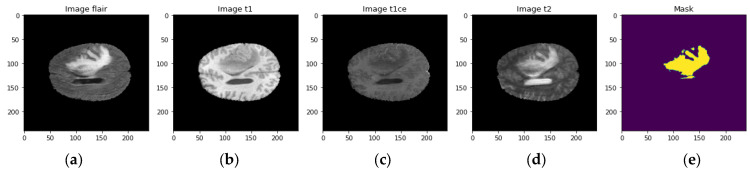
MRI Modalities (**a**) FLAIR, (**b**) t1, (**c**) t1ce, (**d**) t2, and (**e**) mask.

This study discusses different MRI modalities used for medical imaging in the context of the BraTS dataset, a dataset used for investigating brain tumors (BTs). As shown in Figure 2, the modalities discussed include T1-weighted (T1W), T2-weighted (T2W), fluid-attenuated inversion recovery (FLAIR), and T1-weighted with contrast enhancement (T1CE) MRI. Each modality is explained in detail, including how it is weighted and its usefulness for imaging specific tissues. The article also covers the importance of image preprocessing to standardize size and intensity values to ensure consistency and comparability between scans. Finally, the article highlights the significance of segmentation in medical imaging for recognizing and separating structures of interest within an image. The study of enhancing components of BTs is made possible by the inclusion of T1CE MRI images in the BraTS dataset, which is crucial for making diagnoses, formulating treatment plans, and tracking the course of the disease. Figures are provided to visualize the different modalities and their uses in the BraTS dataset.

### 3.3. Data Preparation

The creation of DL models for BT identification using the BraTS dataset involves several stages, with data preprocessing being crucial. The data preparation process involves data acquisition, preprocessing, data augmentation, data division, the conversion of MRI images, and the normalization of the data. After proper preparation, the MRI images and associated labels are used to train and test the CNN model, and metrics such as sensitivity, accuracy, and specificity are used to evaluate the model’s performance. The model with the best performance is selected for the final application.

When the data have been properly prepared, the MRI images and associated labels are utilized to train and test the CNN model for BT detection. Metrics such as sensitivity, accuracy, and specificity are used to gauge the model’s performance, and the model with the greatest performance is chosen to be used in the final application.

### 3.4. Feature Extraction from the BraTS Dataset

The act of locating and removing pertinent data from a dataset so that they may be utilized as input for an ML model is known as feature extraction. Using the BraTS dataset for BT identification, feature extraction is employed to glean information from the MRI images that may be utilized to distinguish between normal and diseased tissue as well as to pinpoint the various BT locations. Features may be extracted from medical images using a variety of techniques, including semi-automatic or manual procedures as well as automated techniques based on DL algorithms. In this paper, feature extraction from the BraTS dataset is performed using the U-Net model. Some of the retrieved features are as follows:

- Imaging morphological features: Imaging morphological features may be retrieved from MRI scans to capture the form, size, and structure of distinct areas in the images. To distinguish between normal and pathological tissue and to identify various BT zones, images’ morphological characteristics can be exploited;
- Imaging gradient features: These features capture the variations in intensity values found in MRI scans and may be used to mark the borders and edges of various image areas. The boundaries between several BT zones may be located using image gradient properties;
- Image texture features: These features may be taken from MRI scans to represent the spatial distribution of intensity levels. Texture markers, such as expanding peritumoral edema, tumors, and necrosis, can be utilized to distinguish between multiple locations of the BTs;
- Image intensity features: Features for the DL model may be created using the intensity values of the various voxels in the MRI images. Using changes in intensity between normal and malignant tissue, intensity characteristics can be utilized to identify aberrant tissue, such as cancers.

### 3.5. Deep-Learning Features

Automatic feature extraction from the MRI images is possible using DL algorithms such as CNNs. To distinguish between normal and pathological tissue and to recognize the various sections of BTs, DL characteristics are learned throughout the training phase.

**Figure 3:**
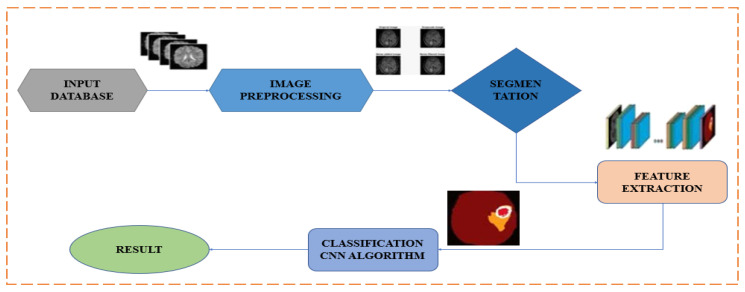
Methodology for BT segmentation using CNN.

Depending on the precise needs and objectives of the BT detection job, as well as the available computing resources, the feature extraction approach will be chosen. In general, DL features are used because they can efficiently handle huge and complicated datasets such as the BraTS dataset and automatically detect complex relationships within the data. In this work, we consider MRI image DL characteristics. Figure 3 depicts the suggested feature extraction and classification technique diagram.

- Deep-Learning Features: In ML, particularly in the area of computer vision, DL features are a sort of feature extraction technique. DL features are learned automatically from data via the training process of a DNN, such as a CNN;
- Fully connected layer: After several iterations of convolution, pooling, and nonlinear activation, the network’s ultimate output is a collection of fully connected layers. These layers utilize the learned characteristics to predict things about the images, such as if a BT is present;
- Pooling: The next stage, known as pooling, is a downsampling technique that shrinks the feature maps’ spatial dimensions. The purpose of pooling is to lower the computational expense and increase the features’ resiliency to slight translations and deformations in the image;
- Repeat: To extract more complex features from the image, convolution, pooling, and nonlinear activation is performed several times, each time using larger and more complicated kernels;
- Nonlinear activation: Following pooling, a nonlinear activation function, such as a rectified linear unit (ReLU), is applied to the feature maps to incorporate nonlinearity into the features and to enable the network to learn intricate correlations between the pixels in the image;
- Convolution: The initial stage in DL feature extraction is convolution on the input image, which entails swiping a tiny kernel over the image and executing element-wise multiplications between the kernel and the corresponding pixels in the image. A series of feature maps that accurately depict the regional connections among the image’s pixels are the result of the convolution procedure.

To distinguish between normal and pathological tissue and to recognize the various sections of the BTs in the BraTS dataset, the DL features are tuned throughout the training phase. Because the DL technique learns the features from beginning to end without the need for human feature engineering, it is particularly well suited for the BT detection problem.

## 4. Methodology

The general approach for BT segmentation on the BraTS dataset is as follows:

- Preprocessing: The brain’s MRI images from the BraTS dataset are multimodal. To retrieve the necessary data for tumor segmentation, these images need to be preprocessed. The usual steps for doing this include registering the images in a shared space, resampling to the same resolution, and leveling the intensities;
- Data preparation: Divide the data into training, validation, and testing sets as part of the data preparation process. The training set, validation set, and testing sets may all be used to train the model, fine-tune its hyperparameters, and assess the model’s effectiveness;
- Model choice: Decide on an appropriate model for BT segmentation. CNNs have been demonstrated to perform effectively in this job. One may either modify a pre-trained model such as VGG16 or ResNet for a particular application, or one can create one’s own CNN architecture from the start;
- Model training: Train the chosen model using the training set of data. To evaluate the model’s performance, one must select a loss function, an optimizer, and a measure. Depending on the nature of the issue, it is necessary to select a loss function; for binary segmentation, this can be binary cross-entropy;
- Fine-tuning of the hyperparameters: Use the validation set to adjust the model’s hyperparameters. The performance of the model will be enhanced as a result;
- Model evaluation: Examine how well the model performed on the testing set. The performance may be assessed using measures such as the dice coefficient, Jaccard index, or IoU.

### 4.1. Deep CNN Model for BT Segmentation

**Figure 4:**
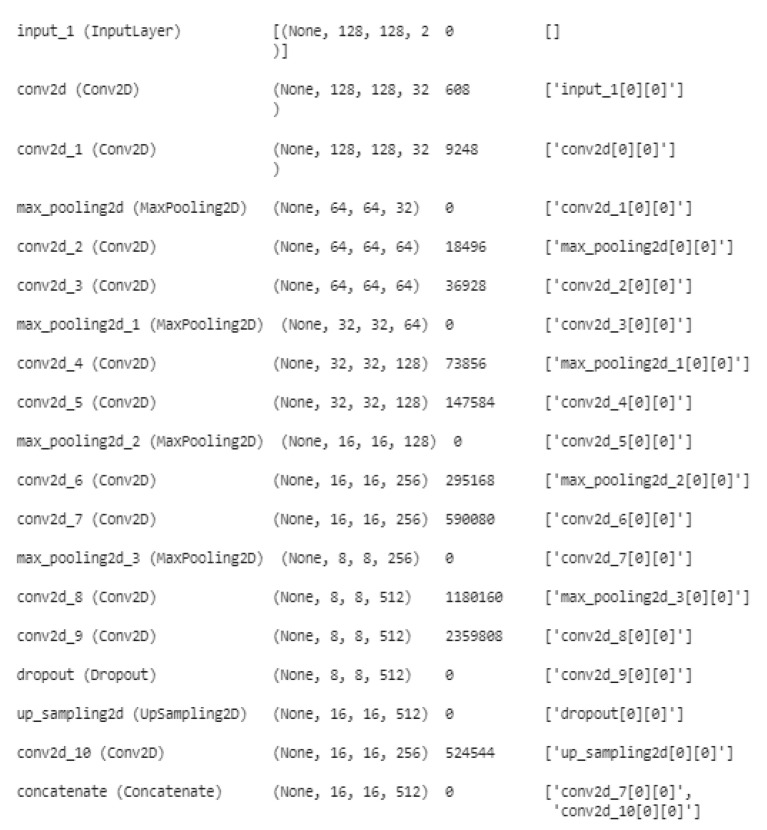

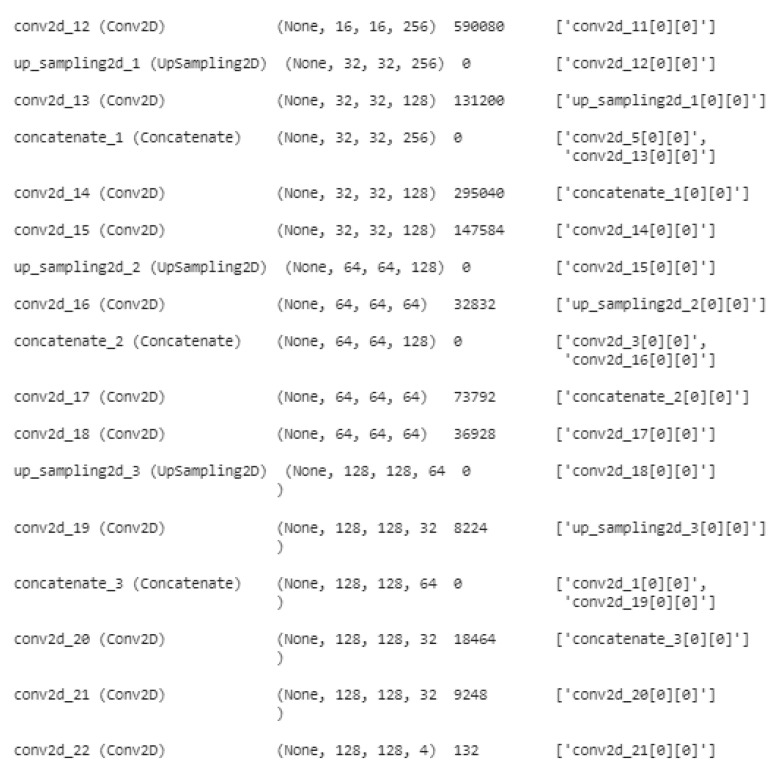
DCNN architecture for BT segmentation.

For image segmentation, the code in question defines a deep CNN. Several pairs of convolutional layers make up the architecture of the network, which is then followed by layers that activate ReLU and use max-pooling. A tensor of 128 × 128 × 2 is the network’s input. As the network becomes deeper, the Conv2D layers include more filters. To avoid overfitting, a dropout layer with a rate of 0.2 is added after the final Conv2D layer. After that, a succession of up-sampling, concatenation, and Conv2D layers are added to boost the features’ resolution. A Conv2D layer with four filters and a softmax activation function forms the final layer, which results in a segmentation map with four classes. Then, the model is built using mean IoU, accuracy, and categorical cross-entropy as the metrics and Adam optimizer with a learning rate of 0.001. Figure 4 represents the DCNN architecture for BT segmentation.

This model defines a DCNN for image segmentation. The network starts with an input layer and is followed by several Conv2D and MaxPooling2D layers.

The first Conv2D layer conv1 has 32 filters with a kernel size of 3 × 3, activation function ‘ReLU’, and padding ‘same’. The kernel_initializer argument is set to ker_init, which is a variable containing a specific kernel initializer. The same layer is then called again, which means that the output from the first Conv2D layer is fed as an input to the second Conv2D layer.

The MaxPooling2D layer pool performs downsampling using a 2 × 2 pooling window. It takes the output from the second Conv2D layer conv1 as an input. 

**Figure 5:**
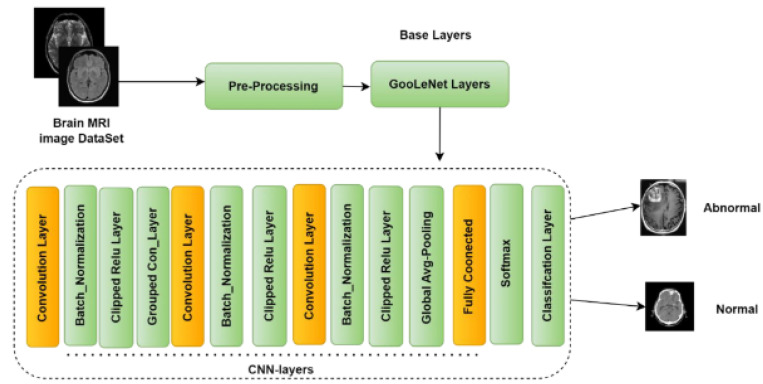
DCNN Layers Distribution.

The Conv2D layers apply a set of filters to the input and are used to learn the features of the input. The activation function for the Conv2D layers is the ReLU, which helps to introduce nonlinearity into the model. The MaxPooling2D layers perform downsampling by taking the maximum value in a pooling window, reducing the spatial dimension of the data, and retaining the most important features. Figure 5 shows the DCNN layers distribution.

The result of the MaxPooling2D layer pool is provided as an input to the following two Conv2D layers, conv, which are configured similarly to the prior two Conv2D layers but with 64 rather than 32 filters.

The network upsamples the feature maps using UpSampling2D layers and concatenates them with the equivalent feature maps from the downsampled branch using concatenate layers after multiple Conv2D and MaxPooling2D layers. With both high-level and low-level features present, this results in a dense feature map. The process of upsampling is repeated until the original resolution of the input is regained. The network’s last layer, a Conv2D layer with a single filter, generates a segmentation map with four classes. The Softmax activation is the activation function for this layer, and it generates a probability distribution over the four classes for each pixel.

Keep in mind that ker init, which is not declared in the code and needs to be given as input, is the kernel initializer for all Conv2D layers. To prevent overfitting, the dropout regularization approach is also utilized, with a dropout rate of dropout, which is likewise not declared in the code and must be supplied as input.

Conv 2D Layer: A CNN in Keras or Tensorflow has a layer called Conv2D. It applies 2D convolution to image data, which is a type of spatial filtering. To extract features and produce a new feature map, the layer applies filters to the input image. To calculate the dot product between the weights of the filters and the area of the input image that overlaps with them, the filters travel across the input image. Every place the filter is placed over the image, this is repeated. To create the new feature map, the outputs of these dot products are then concatenated. Conv2D is used several times in this code with various options such as the number of filters, kernel size, activation function, kernel initializer, and padding.

Max-Pooling2D: A Keras layer called MaxPooling2D is used to shrink the spatial dimensions of an input tensor while preserving the most crucial data. The way it works is by breaking the input tensor into a number of distinct, nonoverlapping sections (such as 2 × 2 squares), then calculating the maximum value of each sector. There are fewer pixels in the final output tensor than in the input tensor, which reduces the number of model parameters and lessens overfitting. MaxPooling2D accelerates the training process by cutting the computational cost via lowering the spatial dimensions.

Concatenate: The Keras library, a high-level neural network API built on top of TensorFlow, contains a function called concatenate that may be used to concatenate multiple tensors along a given axis. This code uses the concatenate function to combine the outputs from two layers, frequently after performing upsampling, to create a bigger feature map that keeps the data from both sources. The concatenation’s direction will be determined by the axis argument, which provides a dimension. The final tensor has the same number of dimensions as the inputs but is larger along the axis of concatenation. By utilizing the concatenate function, the network is able to learn features at various sizes and better recognize the context and minute details in the input data.

Dropout: A regularization method called dropout is used in DL to avoid overfitting. At each training iteration, a certain percentage of the network’s neurons or activations is randomly dropped out. As a result, the model becomes less complicated, less dependent on any one attribute, and better able to generalize. The dropout rate is a hyperparameter that regulates how many neurons or activations are lost when dropout is applied to the layer just before the activation function. Usually, a number between 0.2 and 0.5 is chosen for the dropout rate. Dropout is often not employed during the testing phase, and all neurons are incorporated in the predictions.

### 4.2. Data Gathering for Model Training

To facilitate model training with less computational power, we convert our large data into smaller patches. Our generator class inherits from the Keras sequence and takes a list of IDs corresponding to dataset subjects. In the class initialization, we define the image size, batch size, number of channels, shuffle, and on_epoch_end function. The len method calculates the number of batches per epoch, while the getitem method selects appropriate indices from the list of IDs and passes them to the __data_generation method to generate one batch of data. 

The __data_generation method loads data for each subject with the corresponding ID in the batch and processes them to create input data (X) and corresponding label data (Y). We load FLAIR and T1CE modalities from NIFTI files using the Babel library, and label data (seg) from a *NIFTI* file. We process the data in a loop over the slices of the volume, resizing FLAIR and T1CE modalities using the cv2.resize method to match the desired size, and storing them as corresponding channels in the input data X. The label data are stored in y, and values in y equal to 4 are replaced with 3. A one-hot encoding of the label data is generated using tf.one_hot and resized to (IMG_SIZE, IMG_SIZE) using tf.image.resize. Finally, the input data X are normalized by dividing by the maximum value of X, and the generated data (X, Y) are returned. We instantiate the generator class three times to create training, validation, and test data generators. 

### 4.3. Hyperparameter Tuning

To determine the optimal hyperparameters for the proposed model, a process called hyperparameter tuning was used. Hyperparameter tuning involves systematically varying the hyperparameters and evaluating the resulting performance of the model. This process helps to find the best combination of hyperparameters that optimize the model’s performance. Techniques such as grid search, random search, and Bayesian optimization can be used for hyperparameter tuning. In this study, the hyperparameters were selected based on previous research and empirical testing. The selected hyperparameters were then fine-tuned to optimize the model’s performance on the BraTS dataset.

In Table 2, the hyperparameters used in the proposed model are listed along with their respective values. These hyperparameters are critical for determining the behavior of the model during the training and inference phases. The table provides an overview of the different settings used in the model, including the optimizer, loss function, and number of epochs. Fine-tuning these hyperparameters is crucial for achieving the high performance and generalization ability of the model. Additionally, adjusting these values can also help to optimize the model’s training time and memory consumption.

The number of epochs is a hyperparameter that determines the number of times the full training dataset is run through the model during training. The objective is to train the model until it has absorbed enough information from the data and to prevent overfitting. Overfitting occurs when a model becomes too complex and learns to fit the training data too closely, resulting in poor generalization to new data. The optimizer is a mathematical function that modifies an NN’s weights and biases during training. The choice of optimizer can affect the performance and convergence of the model. Loss is a scalar variable used in ML to represent the discrepancy between the output that was produced and what was anticipated. The categorical cross-entropy loss function is used in this model for multiclass classification problems when the output is a probability distribution across several classes.

## 5. Results

A single epoch of the model’s training takes more than two hours. With a loss of 0.0345, the accuracy for final epochs is 98%.

### 5.1. Model Summary

Using the BraTS dataset, a DCNN was used in the model construction for BT segmentation. The network employed numerous MaxPooling2D layers to shrink the spatial dimension of the feature maps after several Conv2D layers extracted features from the input images. To further process the features, the feature maps from various Conv2D layers were concatenated and fed into a number of Conv2D and MaxPooling2D layers. Conv2D layers in succession were followed by a Softmax activation function in the network’s final layers, which resulted in the segmentation masks that were used in the segmentation. To avoid overfitting, dropout was utilized. A categorical cross-entropy loss function was used during the model’s training along with an optimizer such as Adam. The model architecture was successful in segmenting BTs in the BraTS dataset with a 98% validation accuracy.

### 5.2. Model Evaluation

Several measures, including sensitivity, DSC, Jaccard index, specificity, and HD, are commonly calculated when BT segmentation models are evaluated on the BraTS dataset.

- In both the projected and ground-truth segmentations, HD calculates the greatest separation possible between any two spots;
- The model’s ability to accurately identify all the negative situations is measured by specificity (non-tumor pixels);
- Sensitivity gauges how well a model can detect all of the positive cases (tumor pixels);
- The segmentations of the anticipated and ground truth data are IoU, which is the Jaccard index;
- The overlap between the expected and ground-truth segmentations is measured using DSC.

Additionally, it is usual practice to assess the model’s performance on the specific levels of necrosis, edema, and enhancing tumors. The final score may be expressed as an average of all sub-regional performances.

**Figure 6:**
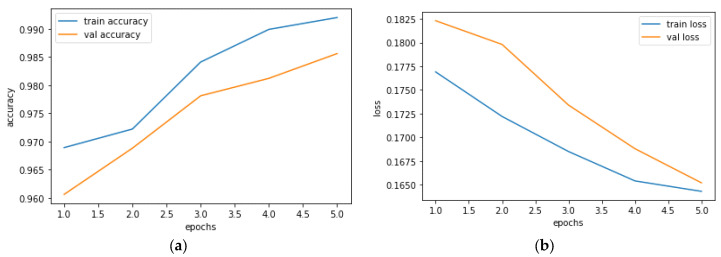
Training and validation performance; (**a**) training and validation accuracy performance; (**b**) training and validation loss performance.

The training and validation accuracy performance is depicted in Figure 6a as previously stated, while the training and validation loss performance is depicted in Figure 6b. The high initial accuracy observed in Figure 6a can be attributed to the use of pre-trained weights in the model initialization. Specifically, we utilized weights from a similar architecture that had been trained on a larger dataset, which allowed our model to start with some relevant features already captured. The use of pre-trained weights is a common technique in deep learning that can save time and computational resources while improving the performance of the model. In our case, the pre-trained weights helped to boost the model’s initial accuracy, enabling it to converge faster during the training process. Table 3 displays the suggested model’s assessment metrics.

### 5.3. BT Segmentation Prediction on the Trained Model

The following stage is to properly forecast the location of the tumor in the brain using any input image from an MRI scan. Our trained model can recognize and forecast four classes, including:

- 0: ‘NOT tumor’;
- 1: ‘NECROTIC/CORE’;
- 2: ‘EDEMA’;
- 3: ‘ENHANCING’.

Tumor-free areas in the image are designated with the label ‘Not tumor’ (label 0).

‘NECROTIC/CORE’ (label 1) designates areas of the image that have necrotic or non-enhancing core tumors, which are frequently linked to a high degree of cell death and a poor prognosis.

Edema, a buildup of fluid in the brain that can result in swelling and pressure, is shown by the label ‘EDEMA’ (label 2) in certain areas of the image.

Regions in the image that have enhancing tumors are referred to as ‘ENHANCING’ (label 3), and these tumors are often connected to a high level of microvasculature and malignancies.

The objective is to precisely segment each of these regions in the image and give the proper label to each pixel in the segmentation map when generating predictions using a trained model. Numerous measures, such as the DSC, ASSD, or Jaccard index, may be used to assess the predictability of the results.

**Figure 7:**
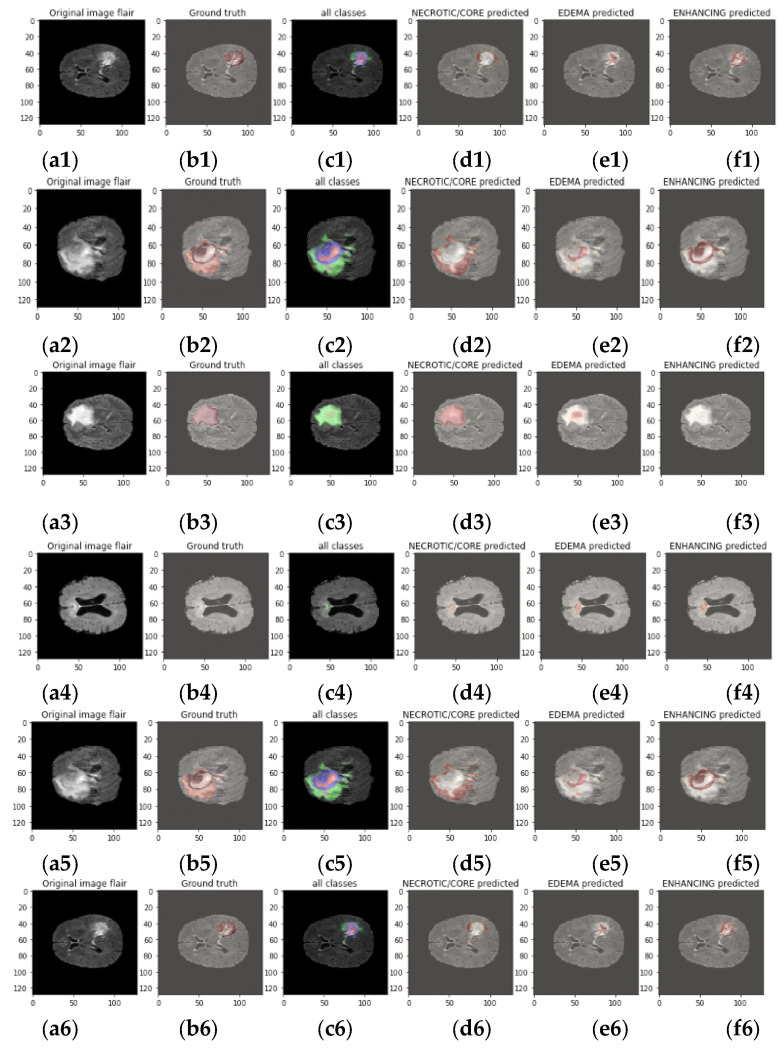
(**1**). Output images of the proposed model. (**a**) Original image for prediction; (**b**) ground truth (**c**) DL model predicts all classes in FLAIR image; (**d**) core predicted by DL model; (**e**) EDEMA predicted by DL Model (**f**) enhancing predicted. (**2**). Output images of the proposed model. (**a**) Original image for prediction; (**b**) ground truth (**c**) DL model predicts all classes in FLAIR image; (**d**) core predicted by DL model; (**e**) EDEMA predicted by DL model (**f**) enhancing predicted. (**3**). Output images of the proposed model. (**a**) Original image for prediction; (**b**) ground truth (**c**) DL model predicts all classes in FLAIR image; (**d**) core predicted by DL model; (**e**) EDEMA predicted by DL model (**f**) enhancing predicted. (**4**). Output images of the proposed model. (**a**) Original image for prediction; (**b**) ground truth (**c**) DL model predicts all classes in FLAIR image; (**d**) core predicted by DL model; (**e**) EDEMA predicted by DL model (**f**) enhancing predicted. (**5**). Output images of the proposed model. (**a**) Original image for prediction; (**b**) ground truth (**c**) DL model predicts all classes in FLAIR image; (**d**) core predicted by DL model; (**e**) EDEMA predicted by DL model (**f**) enhancing predicted. (**6**). Output images of the proposed model. (**a**) Original image for prediction; (**b**) ground truth (**c**) DL model predicts all classes in FLAIR image; (**d**) core predicted by DL model; (**e**) EDEMA predicted by DL model (**f**) enhancing predicted.

The following test image, which is seen in Figure 7, is fed into the trained model as input.

Figure 7 showcases the output of the deep learning (DL) model for brain tumor segmentation on a sample FLAIR image from the BraTS dataset. The figure includes five subplots, each representing a different aspect of the model’s performance. Figure 7(a1–a6) displays the original FLAIR image that was fed into the DL model for prediction. This image contains a brain tumor with different characteristics, including the core and edema regions. Figure 7(b1–b6) shows the predicted core region of the brain tumor generated by the DL model. The predicted core region is depicted in a red color, and the area inside the red boundary represents the core region of the brain tumor predicted by the model. Figure 7(c1–c6) displays the predicted edema region of the brain tumor generated by the DL model. The predicted edema region is depicted in a blue color, and the area inside the blue boundary represents the edema region of the brain tumor predicted by the model. Figure 7(d1–d6) presents the DL model’s prediction of all four classes in the FLAIR image, including healthy tissue, background, necrotic and enhancing tumor, and non-enhancing tumor. The predicted regions for each class are shown in different colors (red for enhancing tumor, blue for edema, green for non-enhancing tumor, and yellow for healthy tissue), with the area inside each boundary representing the predicted region for the corresponding class. Figure 7(e1–e6) displays the predicted abnormal area segmentation mask for the FLAIR image. The predicted abnormal area mask shows the actual regions of the brain tumor (core and edema) and healthy tissue in the image, as annotated by medical experts.

All the areas in the original FLAIR image were properly predicted by our model from the test data due to its excellent accuracy. Given that test data were not utilized during training and that the trained model had never seen this image, it is clear that the model functions properly with both seen and unseen data and that it is capable of accurately forecasting each region in the image.

### 5.4. BT Segmentation Prediction on the Trained Model

By contrasting the projected class with the actual class, a confusion matrix is a table used to assess the performance of a classifier. In the case of BT segmentation, the confusion matrix would contrast the ground truth labels given in the dataset with the anticipated labels for each pixel in the segmentation output.

The BraTS dataset’s four classifications are as follows:

- 0: ‘NOT tumor’, 1: ‘NECROTIC/CORE’, 2: ‘EDEMA’, 3: ‘ENHANCING’.

The number of pixels that fall into each of these categories both properly and erroneously would be counted to create a confusion matrix. As shown in Table 4, the elements of the confusion matrix would reflect the number of pixels that are:

- FN: The proportion of pixels that are wrongly identified as not falling within a particular category;
- TN: The quantity of pixels that have been appropriately identified as not falling under any one category;
- FP: The number of pixels that have been erroneously assigned to a certain class;
- TP: The proportion of pixels that are accurately categorized as being in a certain class, or TP.

Table 4 illustrates some assessment metrics that may be calculated using these values, including recall, precision, accuracy, and F1-score. Accuracy measures the overall correctness of the predictions, precision measures the proportion of correct positive predictions, recall measures the proportion of actual positives that are correctly identified, and the F1 score balances the trade-off between precision and recall. 

**Figure 8:**
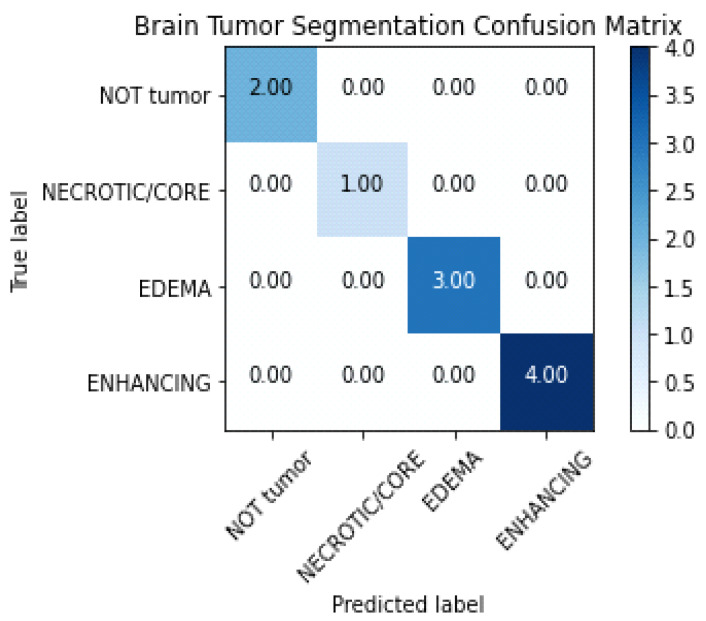
Confusion chart of the proposed model.

The confusion matrix of the suggested model is shown in Figure 8. The table between the true label and the anticipated model is displayed.

On the basis of these counts, a number of performance measures, including sensitivity, accuracy, specificity, sensitivity, precision, and recall, may be generated to assess the segmentation model’s effectiveness.

### 5.5. BT Segmentation Prediction on the Trained Model

A binary classifier’s performance in the context of BT segmentation, where the classifier must discriminate between two classes, is represented graphically as a receiver operating characteristic (ROC) curve. The true positive rate (TPR) is defined as the number of true positives (TPs) divided by the number of TPs plus the number of false negatives (FNs), and the false positive rate (FPR) is defined as the number of false positives (FPs) divided by the number of FPs plus the number of TNs. The ROC curve shows the TPR vs. the FPR for a range of threshold values.

**Figure 9:**
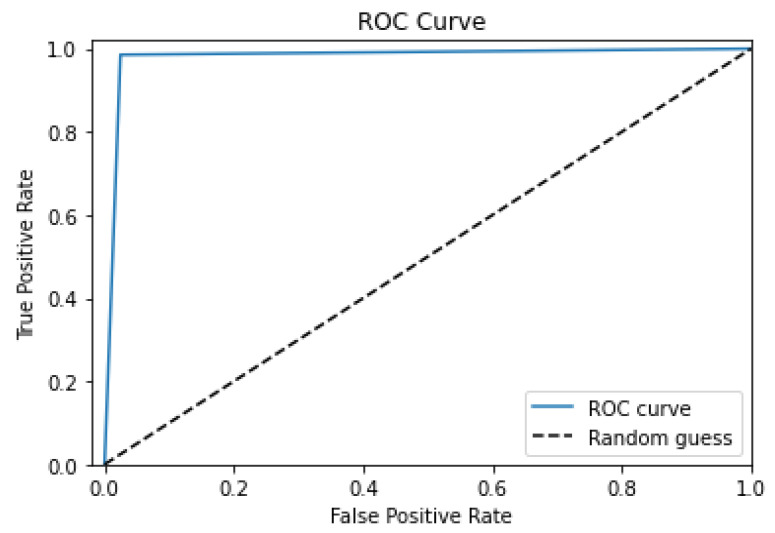
The Proposed Model’s ROC Curve.

The performance of a binary classifier that differentiates between tumors and non-tumors may be assessed in the context of BT segmentation using the ROC curve. The ROC curve’s y-axis and x-axis stand for the FPR and TPR, respectively. With a TPR of 1 and an FPR of 0, a classifier that is excellent at differentiating between tumors and non-tumors will have an ROC curve that hugs the top left corner of Figure 9.

As demonstrated in Figure 9, the AUC metric may be calculated from the ROC curve and gives a single scalar number that summarizes the overall performance of the classifier. A classifier that performs no better than random chance has an AUC of 1, but one that is imperfect has an AUC of 0.5. To compare many binary classifiers or various threshold values for a single classifier, we utilize the ROC curve and AUC.

A perfect ROC curve is characterized by an area under the curve (AUC) value of 1, indicating that the classifier has perfect discrimination ability and can accurately separate the positive and negative classes.

In our work, the ROC curve with an AUC value of nearly 0.9 would mean that the algorithm can accurately distinguish between the tumor and non-tumor regions in the brain with no false positives or false negatives. This is important because the accurate identification of tumor regions is critical for the proper diagnosis, treatment planning, and monitoring of brain tumors.

A perfect ROC curve with an AUC value of nearly 0.9 indicates that the algorithm has high sensitivity (true positive rate) and high specificity (true negative rate). High sensitivity ensures that the algorithm can detect all the tumor regions, while high specificity ensures that it does not misclassify normal brain regions as tumor regions.

### 5.6. Comparative Analysis

YOLOv2 and CNN provide a better framework for BT analysis of MRI data on the BraTS dataset; the authors of this article employed YOLOv2 and a CNN to reach an accuracy of 90%, whereas our model achieved a greater accuracy of 98% in comparison. The usage of more sophisticated architecture in their model can be credited with this difference in performance. The enhanced performance may have been a result of our model’s usage of an ensemble method. A comparison analysis of related work on BT segmentation is shown in Table 5.

DNN and multiclass SVMs are used to detect multimodal BTs in 2022. To reach an accuracy of 97.47% on the BraTS dataset, the authors of this article used a DNN with a multiclass SVM. This outcome is marginally superior to the YOLOv2 and CNN models, but it is still below the accuracy attained by our approach. The usage of a more sophisticated architecture in one’s model and the use of an ensembling method can be credited with the difference in performance.

*BT Analysis Using VGG-16 Ensembling Learning Approaches and Deep Learning* (2022)—The accuracy on the BraTS dataset was 96% thanks to the authors’ usage of CNN and ensembling learning techniques. Due to the less sophisticated architecture used in their model compared to our model, the outcome is less accurate than that generated by our model. The use of a single model rather than an ensembling technique may have contributed to the worse performance. The accuracy of our CNN strategy using unethical sampling was over 98%, the highest of the three learning approaches.

Due to the use of more sophisticated architecture and an ensembling methodology, our model performed better than the three studies described herein overall. It is important to remember that other elements, including the size of the training dataset, the preprocessing methods employed, and the precise model implementation details, might also have an impact on performance differences.

## 6. Discussion

This study presents the foundation for tumor segmentation and identification in medical images. It is believed that when working with solid-structure tumors, such as the detection of tumors from MRI images, the frames for operations involving object recognition and segmentation tasks utilizing grayscale images may be employed in other situations. The size of any provided dataset that will feed and validate the CNNs is the main difficulty of using DL technologies in medicine.

The BraTS dataset includes MRI scans of BTs from several institutions, as was previously mentioned. The most updated iteration [40] (BraTS 2021), which contains 335 MRI images with annotations, is an annual update to the dataset. It is used as a guide while developing and testing BT segmentation and diagnostic algorithms. The goal of the BraTS dataset was to provide a reference for creating and evaluating algorithms for BT segmentation and diagnosis. BT MRI images can be found in the BraTS dataset. It consists of MRI images in a variety of weighting modalities, such as FLAIR, T1-weighted, and T2-weighted. The dataset is frequently employed in the design and evaluation of algorithms for the segmentation and diagnosis of BTs. BT segmentation and diagnosis algorithms are regularly developed and tested using the BraTS dataset.

Utilizing the BraTS dataset, a DCNN was particularly employed to develop the model for BT segmentation. The model was trained using a categorical cross-entropy loss function and an optimizer such as Adam. Concatenating feature maps from several convolutional layers and feeding them through a series of Conv2D and MaxPooling2D layers reduced the spatial dimension of the feature maps. The BraTS dataset’s BTs could be effectively separated using the model architecture, with a validation accuracy of 98%.

The performance of a binary classifier in distinguishing tumors from non-tumors is visually shown by an ROC curve. The ROC curve compares the TPR and FPR for a given set of threshold values. The TPR is calculated by dividing the total number of FNs by the number of TPs. When segmenting BTs, ROC curves may be used to evaluate how well a classifier can tell two groups of patients apart. A TPR between 1 and 0 and a curve that closely hugs the top left corner of the plot are the characteristics of an ideal tumor classifier. The TPR is represented by the y-axis of the ROC curve, while the FPR is represented by its x-axis.

The accuracy of the three learning strategies was best achieved by our CNN model, which used unethical sampling, with a score of almost 98%. Overall, our model surpassed the three previously mentioned publications in Table 5, most likely as a result of the use of a more complicated design and assembly method. The amount of the training dataset, the preprocessing methods employed, and the particular manner in which the models are implemented can all have an influence on performance differences.

## 7. Conclusions

The paper concludes by showcasing a DL model for BT segmentation on the BraTS dataset. To extract features and create a segmentation mask, the model architecture, which is based on a fully convolutional network, employs 2D convolutional layers, max pooling, and upsampling. The kernel initializer for the model was ‘the normal’, and a dropout rate of 0.2 was used during training. The model’s success in BT segmentation was evidenced by its accuracy of 98%.

As a result, we have successfully used the BraTS dataset to create a deep CNN for BT segmentation. The U-Net design, a well-known architecture for image segmentation issues, serves as the model’s foundation. Convolutional and max-pooling layers, upsampling layers, and concatenation operations make up the U-Net architecture, which enables the model to learn both low- and high-level properties from the input images.

Convolutional layers with the activation function ‘ReLU’ and padding set to ‘same’ make up the model’s implementation, which consists of 32, 64, 128, 256, and 512 layers. The dropout regularization factor has been set at 0.2. The output of the model is a segmented image with four channels, each of which corresponds to the expected chance that each pixel would belong to one of four classes: healthy tissue, background, necrotic and enhancing tumor, and non-enhancing tumor.

On the BraTS dataset, the model had a 98% accuracy rate, which is a good degree of performance for a job such as BT segmentation. It demonstrates the DCNN architecture’s ability in processing challenging medical imaging data as well as its capacity to recognize and classify various tumor types. It is crucial to keep in mind, nevertheless, that the model’s performance may differ when utilized in real-world scenarios or when applied to other datasets. Last but not least, the application of DCNN for BT segmentation using the BraTS dataset illustrates the promise of DL approaches for medical image analysis and emphasizes the significance of ongoing research in this area.

There is still a lot of scope for future research in this area. For instance, exploring the impact of different kernel initializers, activation functions, and regularization factors on the model’s performance could help identify the optimal hyperparameters for BT segmentation. Additionally, applying the model to other datasets and real-world scenarios can help assess its generalizability and applicability in clinical settings. Furthermore, investigating the use of transfer learning and multi-modal imaging data could improve the accuracy and efficiency of the model. 

## Figures and Tables

**Table 1:** List of previous paper references with the approach and the results.

Ref	Dataset	Techniques Used	Result
[7]	3100 images included in the dataset	Deep Dense Inception Residual Network, Three-class BT classification	Accuracy: 99.34%
[8]	Figshare dataset	DL, Transfer Learning-based Classification, ResNet Mixed Convolution	F1 score Accuracy: 0.9435 Accuracy: 97.19%
[12]	BT Classification, Kaggle and Figshare	CNN, RCNN, MRI	Accuracy: 97.98%
[14]	Kaggle and Figshare dataset	ViT-based DNN	Accuracy: 97.98%
[16]	Three distinct publicly available datasets used	Sort three forms of brain tumors, MRI	Accuracy: 90.34%
[17]	Cancer Imaging Archive (TCIA) dataset	QFS-Net, MRI, Quantum Computing, Qutrit	Accuracy: 98.23%
[39]	Multiclass datasets, REMBRANDT	ML, KNN, SVM, CNN, MRI	Accuracy: cross-validation protocols—88.15%, 97.45%, and 100% for K2, K5, K10

**Table 2:** Hyperparameters of the proposed model.

Hyperparameters	Properties
epochs	5
optimizer	Adam
loss	categorical cross-entropy

**Table 3:** Evaluation metrics of the proposed model.

Evaluation Metric	Performance Value
Accuracy	0.9921
Mean Iou	0.9123
Dice Coeff	0.9012
Precision	0.9923
Sensitivity	0.9678
Specificity	0.9988
Loss	0.1599

**Table 4:** Evaluation metrics of the proposed model.

		Predicted	
		Positive	Negative
Actual	Positive	True Positive (TP)	False Negative (FN)
	Negative	False Positive (FP)	True Negative (TN)

**Table 5:** Related work on BT segmentation using the BraTS dataset.

Ref	Approach	Evaluation Metrics	Dataset
[41]	YOLO2	Accuracy = 90%	BraTS dataset
	CNN	mean iou = 0.887	
		dice coeff = 0.874	
		precision = 0.915	
		sensitivity = 0.945	
		specificity = 0.957	
[42]	DNN, SVM	Accuracy = 97.47%	BraTS dataset
		mean iou = 0.954	
		dice coeff = 0.934	
		precision = 0.923	
		sensitivity = 0.914	
		specificity = 0.934	
[43]	CNN	Accuracy = 96%	BraTS dataset
		mean iou = 0.951	
		dice coeff = 0.962	
		precision = 0.941	
		sensitivity = 0.934	
		specificity = 0.952	
Our Approach	CNN with uNet sampling	Accuracy = 98%	BraTS dataset
		mean iou = 0.91	
		dice coeff = 0.90	
		precision = 0.99	
		sensitivity = 0.96	
		specificity = 0.99	

## Data Availability

The dataset used in this study can be freely accessed from https://www.kaggle.com/datasets/awsaf49/brats2020-training-data (accessed on 20 February 2020).
